# Fitbit wear-time and patterns of activity in cancer survivors throughout a physical activity intervention and follow-up: Exploratory analysis from a randomised controlled trial

**DOI:** 10.1371/journal.pone.0240967

**Published:** 2020-10-19

**Authors:** Sarah J. Hardcastle, Ruth Jiménez-Castuera, Chloé Maxwell-Smith, Max K. Bulsara, Dana Hince

**Affiliations:** 1 School of Health and Human Performance, Dublin City University, Dublin, Ireland; 2 Institute for Health Research, University of Notre Dame, Fremantle, Western Australia, Australia; 3 Faculty of Sport Sciences, University of Extremadura, Cáceres, Spain; 4 School of Psychology, Curtin University, Bentley, Perth, Western Australia, Australia; Linneaus University, SWEDEN

## Abstract

**Objective:**

There has been growing interest in the use of smart wearable technology to promote physical activity (PA) behaviour change. However, little is known concerning PA patterns throughout an intervention or engagement with trackers. The objective of the study was to explore patterns of Fitbit-measured PA and wear-time over 24-weeks and their relationship to changes in Actigraph-derived moderate-to-vigorous PA (MVPA).

**Methods:**

Twenty-nine intervention participants (88%) from the *w*earable *a*ctivity *t*echnology *a*nd *a*ction-*p*lanning (WATAAP) trial in colorectal and endometrial cancer survivors accepted a Fitbit friend request from the research team to permit monitoring of Fitbit activity. Daily steps and active minutes were recorded for each participant over the 12-week intervention and throughout the follow-up period to 24-weeks. Accelerometer (GT9X) derived MVPA was assessed at end of intervention (12-weeks) and end of follow-up (24-weeks).

**Results:**

Fitbit wear-time over the 24-weeks of data was remarkably consistent, with median adherence score of 100% for all weeks. During the intervention, participants recorded a median 8006 steps/day. Daily step count was slightly increased through week-13 to week-24 with a median of 8191 steps/day (*p* = 0.039). Actigraph and Fitbit derived measures were highly correlated but demonstrated poor agreement overall. Fitbit measured activity was closest to MVPA measured using Freedson cut-points as no bias was observed.

**Conclusions:**

Step count was maintained throughout the trial displaying promise for the effectiveness of smart-wearable interventions to reduce sedentary behaviour beyond the intervention period. Further worthwhile work should compare more advanced smart-wearable technology with accelerometers in order to improve agreement and explore less resource-intensive methods to assess PA that could be scalable.

## Introduction

Despite the established health benefits of regular PA for cancer survivors including reduced cardiovascular disease [1], mortality [2, 3], cancer recurrence [2], and improved quality of life [4], many fail to meet the PA guidelines of at least 150-minutes of moderate-intensity exercise [5]. Wearable technology interventions show promise in the development of low-cost and scalable approaches to promote PA and reduce sedentary behaviour. Reviews of trials incorporating wearable activity trackers support their effectiveness for increasing PA [6] and reducing sedentary behaviour [7]. There is evidence to support the effectiveness of wearables for increasing PA amongst adults with chronic disease [8], post-menopausal women [9], and in breast cancer survivors [10, 11]. However, despite the active and growing research on ‘smart’ wearables (rather than pedometers) to promote PA, little is known concerning patterns of PA throughout an intervention.

Self-monitoring interventions have successfully increased PA in cancer survivors [12], and survivors report self-monitoring to be helpful for increasing PA [13, 14]. Self-monitoring is an evidence-based behaviour change technique that has demonstrated success in the promotion of healthy eating and physical activity [15, 16] in addition to weight management [17]. Smart wearable activity trackers may offer superior self-monitoring compared to paper and pencil techniques and older pedometers given their real time feedback automatic prompts. Lyons et al [18] reviewed 13 different wearable trackers and their associated apps and concluded that they use many behaviour change techniques employed in typical PA interventions (i.e. self-monitoring, feedback, goal-setting). The behaviour change techniques that are associated with greater effectiveness in PA interventions include self-monitoring, goal-setting, review of goals, and feedback on performance [15]. Smart wearable trackers and their associated Applications (Apps) provide these evidenced-based techniques and therefore offer a potentially cost-effective and scalable approach to PA promotion.

Despite the promise of smart wearable trackers, to date, few studies have explored engagement with trackers or patterns of PA throughout a behaviour change intervention. Engagement is important given the behaviour change techniques provided through tracker and App. Further, review level evidence suggests that regular use of a pedometer is associated with greater PA participation [19].

Cadmus-Bertram and colleagues conducted a thorough assessment of Fitbit engagement over a 16-week intervention with obese menopausal women and found sustained engagement over the intervention and stable activity levels [20]. Hartman and colleagues [21] also examined patterns of Fitbit use in breast cancer survivors and activity in a 12-week intervention including associations with success in the intervention [21]. The authors reported high and stable Fitbit adherence and that greater adherence to wearing the Fitbit was associated with greater increased in Actigraph-measured MVPA. Both previous studies explored engagement over a relatively short time period and did not assess tracker engagement during a follow-up period (i.e., once intervention support is removed). Given concerns regarding sustained engagement with wearable activity trackers [22], further work is needed to explore sustained use of trackers and patterns of PA over time and, once intervention supports have been removed. To our knowledge, this is the first study to explore wearable tracker wear-time throughout the intervention and maintenance period.

The aim of this study was to explore data collected from the Fitbit device weekly for 24-weeks in a sample of colorectal and endometrial cancer survivors enrolled in the Wearable Activity Technology And Action-Planning (WATAAP) trial. The primary aims were: (i) explore Fitbit wear-timr throughout the trial and between the intervention phase (to 12-weeks) and the follow-up period (week-13 to week-24); (ii) assess the association between Fitbit wear-time and changes in Actigraph-measured MVPA; (iii) examine patterns of physical activity (steps and active minutes) derived from the Fitbit, particularly in relation to contact with study personnel; and (iv) examine the relationship and agreement between Fitbit-measured active minutes and Actigraph-derived MVPA at 12-weeks. We hypothesised that greater Fitbit wear-time would be associated with greater increases in Actigraph-measured MVPA.

## Methods

### Participants and design

Participants in this secondary exploratory analysis were enrolled in the WATAAP trial [23]. The study was granted approval by the St John of God Healthcare Human Research Ethics Committee (reference # 1102). Written informed consent was obtained from participants prior to enrolment.

Eligible participants included stage 1 or 2 colorectal or endometrial cancer survivors who had completed active cancer treatment within the five-years prior to recruitment and were deemed insufficiently physically active (defined as completely less than 150-minutes of MVPA [5] and at CVD risk (i.e., overweight or obese, hypertensive or hypercholesteraemic). The full eligibility criteria have been previously published [23]. Exclusion criteria included a current diagnosis of a severe psychiatric illness or cardiac abnormalities, severe disabilities including arthritis, already enrolled in a PA program/trial and/or already using a wearable tracker.

Of the 471 patients invited by mail to participate in the study, 109 expressed interest and were screened for eligibility. Many (*n* = 345) did not reply to the invitation and 17 declined. Reasons for declining included personal reasons (*n* = 11), travel difficulties (*n* = 3) and failure to respond following initial contact (*n* = 3). Of the 109 screened for eligibility, 41 failed to meet the inclusion criteria. The most common reasons for being ineligible included active cancer (*n* = 14), sufficiently active (n = 7), already tracking activity (*n* = 5), upcoming surgery (*n* = 4), away during study (*n* = 4), and physical impairments (*n* = 3). A total of 68 participants attended the baseline assessment and were randomized to the intervention group (*n* = 34) or the control group (*n* = 34). Three participants from the control arm and one participant from the intervention group dropped out of the study resulting in a 94% retention rate. The present analyses include data from the 33 participants who were randomized to the intervention group and completed the study. The participants began the 12-week intervention on the 18^th^ September 2017 through to 10th December.

The study protocol and main outcomes have been published elsewhere [23, 24]. In brief, eligible survivors were identified from oncologists’ medical records and were mailed a participant information sheet and invitation letter. Individuals who expressed interest were screened by telephone to ensure eligibility. Those eligible attended an in-person baseline assessment where they were given an Actigraph GT9X accelerometer to wear for the following 7-consecutive days and mail back to the research team in the pre-paid satchel. Following baseline assessments, an independent statistician who was blinded to the assessments and intervention, randomized participants using consecutive randomization codes (STATA v14) with a 1:1 allocation in blocks of 4. The trial co-ordinator contacted those assigned to the intervention to invite them to the first group meeting where participants received a Fitbit Alta. Assessments post-randomization (at 12-weeks, immediately following intervention) and at 24-weeks (follow-up) were conducted at St. John of God Subiaco Hospital by hospital staff blinded to group allocation. Participants were given an Actigraph Link GT9X accelerometer, waistband, log, and postage materials. Participants were instructed to wear the accelerometer on the day following their assessment and continue wear for seven consecutive days before posting the accelerometer back to the research team.

### Intervention

The 12-week intervention consisted of three components, which have previously been described [23]. The primary tool was the provision of a Fitbit Alta, a wrist-worn tracker with demonstrated acceptability for use in cancer survivors [25]. The role of the Fitbit was to offer continuous self-monitoring and track steps and ‘active minutes’ (MVPA accrued in bouts of ≥10-minutes). It also targets sedentary behaviour by providing automated prompts encouraging participants to accumulate ≥250 steps/hour. Participants received and set-up their Fitbit during the first group session (week 1). Any problems with setting up the Fitbit or syncing were identified early in the intervention and dealt with at the second group session at week 4 or between week 1 and week 4.

Participants attended two, two-hour group sessions (~11 per group) facilitated by behaviour change specialist SJH and CMS in weeks 1 and 4. Session 1 involved Fitbit set-up and a presentation concerning PA messaging, instruction on performance of the behaviour, goal-setting, confidence building, action-planning, and self-monitoring of active and sedentary behaviour. Emphasis was given to reducing bouts of sedentary behaviour and responding to the automatic prompts to take steps, in addition to planning deliberate bouts of MVPA. Participants were assisted to complete action-planning and goal-setting activities. Session-2 focused on reviewing goals, forming ‘if-then’ plans to overcome barriers, based on our previous research [13, 26]. Home-based strength exercises were demonstrated with an opportunity for practice and participants were encouraged to log strength training manually on the Fitbit app. During week 8, participants received a 20-minute phone-call to provide support and feedback regarding PA progress, review goals, action plans and coping-planning strategies. Intervention contact ended at 8-weeks and following the assessment at 12-weeks (T2), a further 12-weeks elapsed to explore self-regulation of PA without any formal support.

### Measures

The Fitbit Alta was used to explore patterns of PA throughout the 12-week intervention (week 2 to week 12) and follow-up period (week 13 to week 24). Fitbit wear-time was collected manually for each participant via the Fitbit application. A step count of ≥1000 steps per day was defined as a valid wear-day. This definition for valid-wear was used because some participants chose to focus on step count rather than MVPA and may not accumulate minutes of MVPA. Further, participants were not instructed to wear the Fitbit all day (albeit encouraged to), and therefore a minimum of 1000 steps/day or more was denoted as valid-wear. Active minutes were derived from the calculation that Fitbit provides and is based on metabolic equivalents (METs). This metric is used to estimate exercise intensity. Active minutes are earned from activities that are at or above 3 METs (i.e., the threshold for moderate intensity physical activity). In alignment with the Center for Disease Control's “10 minutes at a time” concept, minutes are only awarded after 10 minutes of continuous MVPA [27].

The Actigraph GT9X research grade accelerometer provided minutes/week of MVPA at baseline (pre-randomisation), T2 (immediately following intervention at 12-weeks) and T3 (follow up at 24-weeks). Participants wore the accelerometer on their right hip for all waking hours across seven consecutive days at each assessment time point. Wear time had to exceed ten hours per day for at least 5 days and contain no excessive counts (>20,000) to be considered valid, with non-wear time defined as at least 60-consecutive minutes of zero counts [28]. Data were processed using 60-second epochs. Daily accelerometer logs were completed by participants to allow for cross-checking of data. Both uniaxial (Freedson) [29] and triaxial (Sasaki) [30] cut points were used to define MVPA. MVPA was defined as ≥1952 and ≥2690 counts per minute for uniaxial and triaxial definitions of cut points respectively [29, 30].

### Data analysis

Demographic characteristics (mean and SD) of the intervention group at baseline were calculated. Seven-day averages were calculated for Fitbit-recorded steps per day and ‘active minutes’ per day for each week of the 24-week trial. Where seven days of data were unavailable, the averages were based on the available data. Fitbit wear-time (as a proxy for engagement) was measured by calculating the percent of days in the first 12-weeks (intervention period) and follow up period (week 13 to 24) that the participant had a valid-wear day (≥1000 steps/day). Changes in Actigraph-measured MVPA and MV10 (MVPA performed in bouts of ≥10 consecutive minutes), for both uniaxial and triaxial definitions, were calculated as the difference between week-12 and week-2 (Phase 1: intervention) and week-24 and week-12 (Phase 2: follow-up) for each activity measure.

As there was a high degree of left skew in activity wear-time measures, these were summarised using medians and interquartile ranges. The Wilcoxon Sign Rank test was used to compare percentage Fitbit wear-time between study intervention phase and follow-up phase. Spearman’s rank order correlation coefficient was used to measure the relationship between Fitbit wear-time and changes in Actigraph measured activity within each phase.

In order to assess the impact of contact with study personnel on steps and active minutes derived from the Fitbit across the 24 week course of the study, Wilcoxon Sign Rank tests (using the normal approximation of this test statistic) were conducted comparing these measures during the week of contact (week 4, 8, 12) to that of the week before and after for both activity measures.

Only participants who had valid data from both the Fitbit concomitant with wearing the Actigraph were included in the device comparison analyses. Spearman’s rank order correlation was used to assess the strength of the association between activity measured on the two devices. The pattern of agreement between the Actigraph-defined MVPA (uniaxial and triaxial), MV10 (uniaxial and triaxial) and Fitbit defined active minutes was explored using Bland Altman plots [31]. All analyses were conducted in Stata v16.1 and *p* < 0.05 was considered significant.

## Results

Most participants (88%, *n* = 29) in the intervention group agreed for their Fitbit data to be collected. One participant experienced syncing errors and was excluded from further analyses. Participants in this secondary analysis (*n* = 28) were an average 65 years old (SD 7.0) and 59% were female. The majority (72.4%) were colorectal survivors. Participants were mostly overweight (41%, *n* = 12) or obese (34.5%, *n* = 10). The median total number of minutes of moderate-to-vigorous physical activity (using uniaxial cut points) at baseline for the sample was 200 mins/week (IQR: 117–242). Eighteen participants (64%) were achieving the guidelines of 150 mins/week of MVPA at baseline. Demographic characteristics of the sample are presented in Table 1. There were no differences in demographic variables or physical activity between those included in the present analysis and the participants in the intervention group that did not provide access to Fitbit data except for relationship status. Participants that did not provide access to their Fitbit data were more likely to be single (X² (4) = 11.063, p = .026).

**Table 1:** Baseline characteristics of participants.

	N = 28	Mean (SD)/N (%)
Age (mean, *SD*)		65.0 (7.0)
Gender		
	Female	17 (58.6%)
	Male	12 (41.4%)
Marital status		
	Married/In a relationship	21 (72.4%)
	Divorced/Separated	6 (20.7%)
	Single	1 (3.4%)
	Widowed	1 (3.4%)
Education		
	University degree	15 (51.7%)
	Post-school training	8 (27.6%)
	High school	6 (20.7%)
Household income (AUD)		
	≤$30,000	4 (13.8%)
	$30,001-$52,000	8 (27.6%)
	$52,001-$104,000	7 (24.1%)
	$104,001-$156,000	5 (17.2%)
	$156,001-$208,000	1 (3.4%)
	$208,001 +	3 (10.3%)
Smoking status		
	Non-smoker	21 (72.4%)
	Ex-smoker	7 (24.1%)
	Current smoker	1 (3.4%)
Comorbidites		
	Overweight	22 (75.9%)
	Obese	11 (37.9%)
	Hypertensive	20 (69.0%)
	Hypercholesterolemic	6 (20.7%)
	Diabetic	3 (10.3%)
	Insufficiently active	27 (93.1%)
Cancer type		
	Colorectal	21 (72.4%)
	Gynaecologic	8 (27.6%)
Treatment		
	Surgery only	12 (41.4%)
	Surgery with chemotherapy	10 (34.5%)
	Surgery with radiation therapy	2 (6.9%)
	Surgery with chemotherapy and radiation therapy	5 (17.2%)

*Note*. Hypertensive: ≥140/90mmHg or taking antihypertensive medication. Hypercholesterolemic: total cholesterol >5.2mmol/L or taking antihypercholesterolemic medication. Insufficiently active: completing <150-minutes/week of MVPA.

### Fitbit wear-time and relationship to changes in Actigraph measured activity

**Fig 1:**
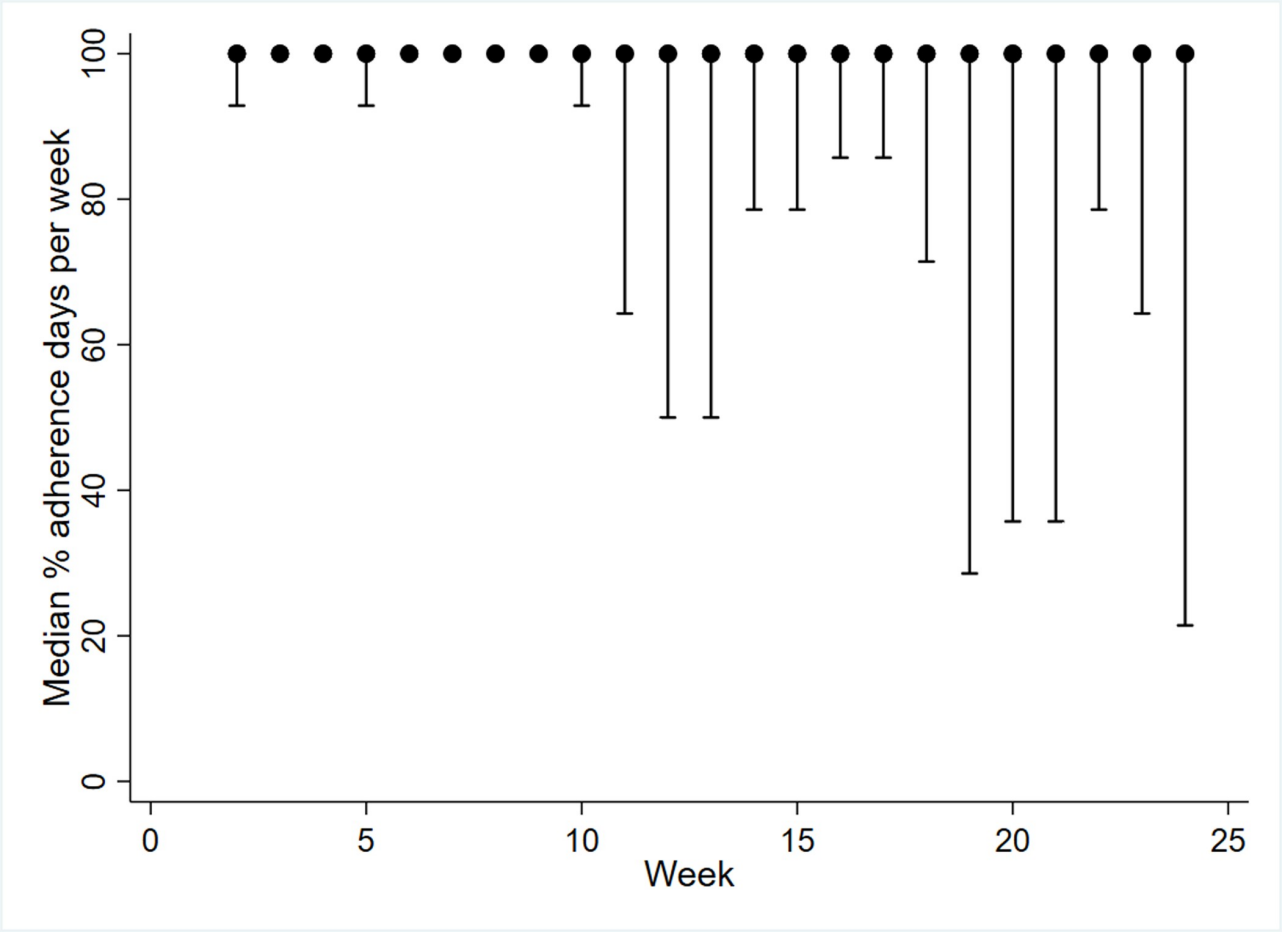
Median percent adherence days per week for week 2–24. The error bars represent the lower bound of the interquartile range (25^th^ percentile). The median value and upper bound (75^th^ percentile) was 100% (7/7 days) for all weeks.

Fitbit wear-time (percentage of valid wear days) over the 24 weeks of data collection was remarkably consistent, with median adherence score of 100% for all 24 weeks considered individually, with the widest IQR occurring at week 24 (21 to 100%) and the narrowest at weeks 3, 4, and 5 to 9 (100 to 100%). There was some variation between participants, with all but week 2 ranging from 0 to 100% (see Fig 1). Fitbit wear-time over the intervention period (week 2 to week 12) was high, with a median of 99% (IQR 88 to 100). Fitbit wear-time was also high during the follow-up period (13-weeks to 24-weeks) with a median adherence score of 98% (IQR 75 to 100). The small difference in adherence rates between the two phases was not significant (z = 1.69, *p* = 0.093). Four participants did not appear to engage with their Fitbit beyond week four.

**Fig 2:**
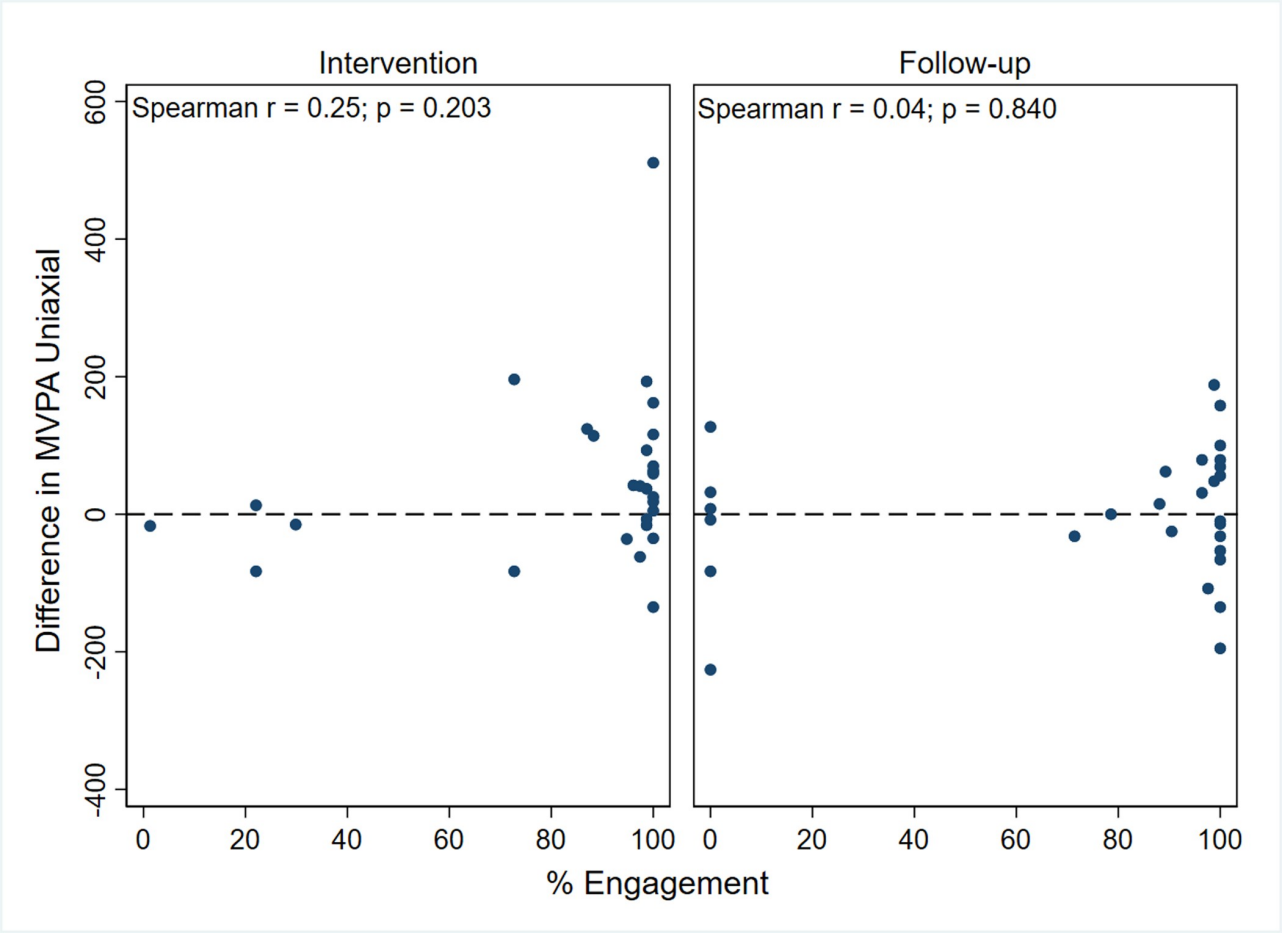
Adherence to Fitbit and change in Actigraph measured moderate-to-vigorous physical activity using uniaxial cut points. Change in Actigraph measured Moderate-to-Vigorous Physical Activity (MVPA) across the intervention period (week 2 to 12) and follow up period (week 12 to 24) as a function of percentage engagement with the Fitbit over the same period. R is Spearman’s rank order correlation.

Fig 2 displays the relationship between percent adherence to the Fitbit and the change in Actigraph measured uniaxial MVPA for the intervention and follow-up phases of the study. Spearman’s rank order correlations is also shown in the figure. No associations were found between these measures, or between MVPA triaxial and MV10 uniaxial and triaxial Actigraph measures and percent adherence during intervention and follow-up phases (S1 Fig). Spearman’s r ranged between 0.33 and -0.21, and none were significantly different from 0.

### Changes in patterns of Fitbit recorded steps and MVPA over time

During the intervention period (week-2 to week-12), participants recorded a median 8006 steps/day (IQR 6996 to 10993) and 229 active minutes/week (IQR 145 to 319) as recorded by Fitbit. Daily step count was slightly increased through week-13 to week-24 with a median of 8191 steps/day (IQR 7302 to 10365; z = 2.1; *p* = 0.039). However, active minutes was lower in the second half of the trial with a median of 210 active minutes/week (IQR 161 to 303) (z = 1.9; *p* = 0.053).

**Fig 3:**
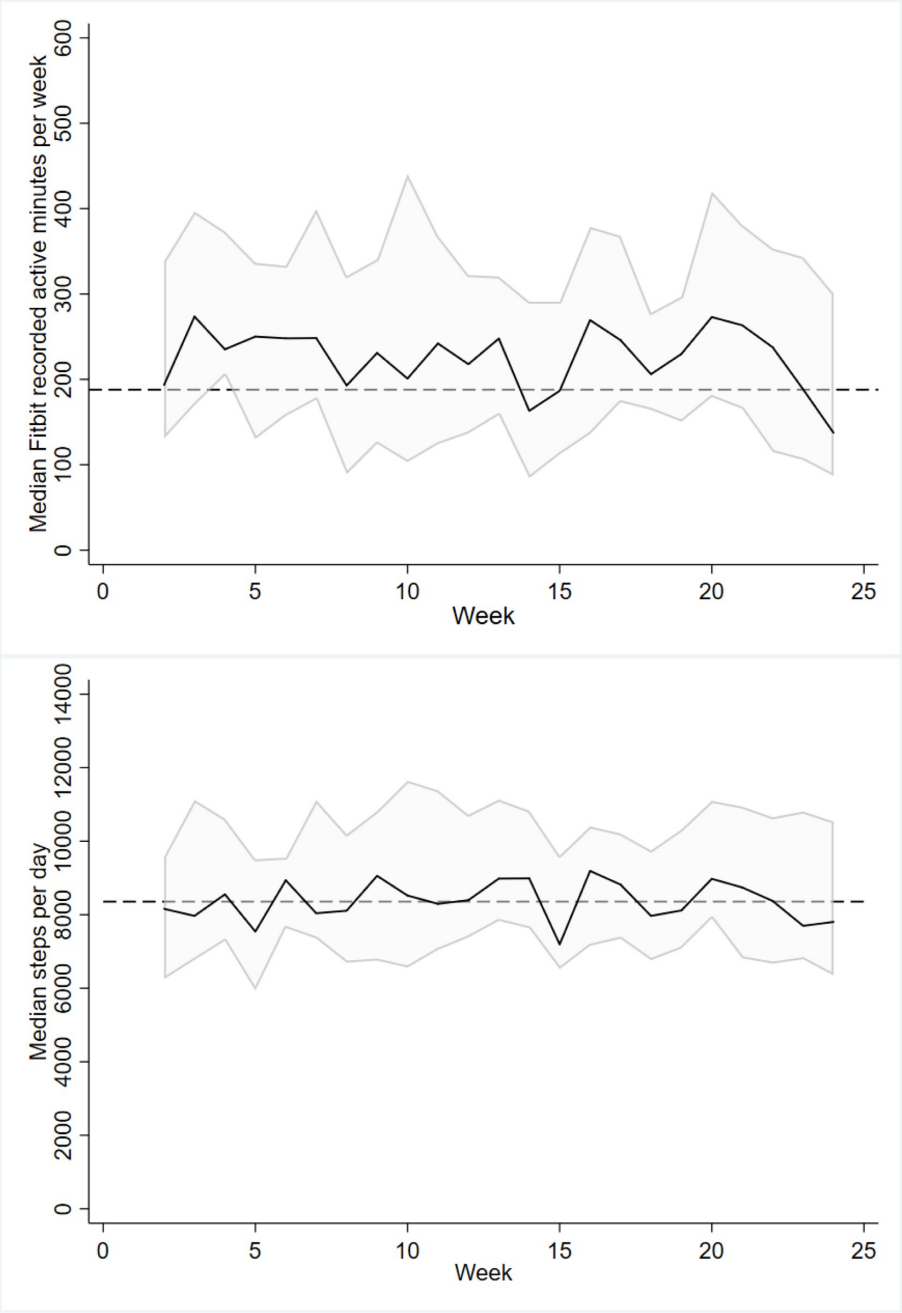
Fitbit median steps per-day per-week and median active minutes per-week across the trial. The shaded error represents the interquartile range around the median (thick black line). The dotted line represents the median steps per day and median active minutes for the duration of the study, respectively.

Median Fitbit measured steps and active minutes per week for the duration of the study are shown in Fig 3. In order to assess whether contact with study staff impacted upon steps and active minutes during each contact week (week 4, 8 and 12) were compared to that of the previous and following week. The difference in medians between the weeks were small, and no significant differences were noted (see S1 Table).

### Device comparison at week 12

**Fig 4:**
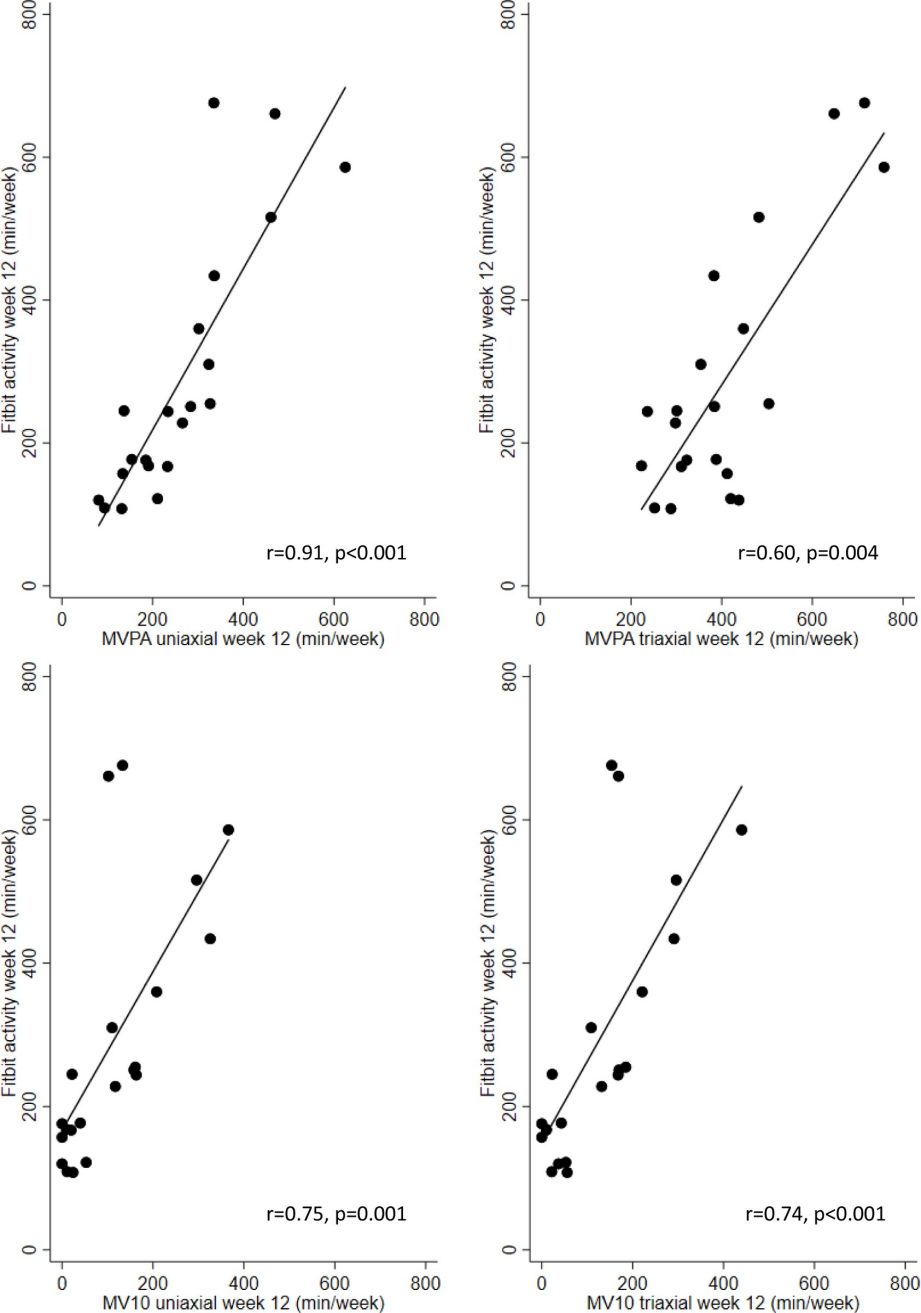
Association between Fitbit active minutes and Actigraph derived moderate-to-vigorous physical activity. Scatter plots for Fitbit and Actigraph Moderate-to-Vigorous Physical Activity (MVPA) (top) and Fitbit and Actigraph MVPA performed in ≥10-minute bouts (MV10) (bottom) measured activity for uniaxial (left) and triaxial (right) cut points. The diagonal line represents the line of best fit for these data. r is Spearman’s rank order correlation coefficient.

**Fig 5:**
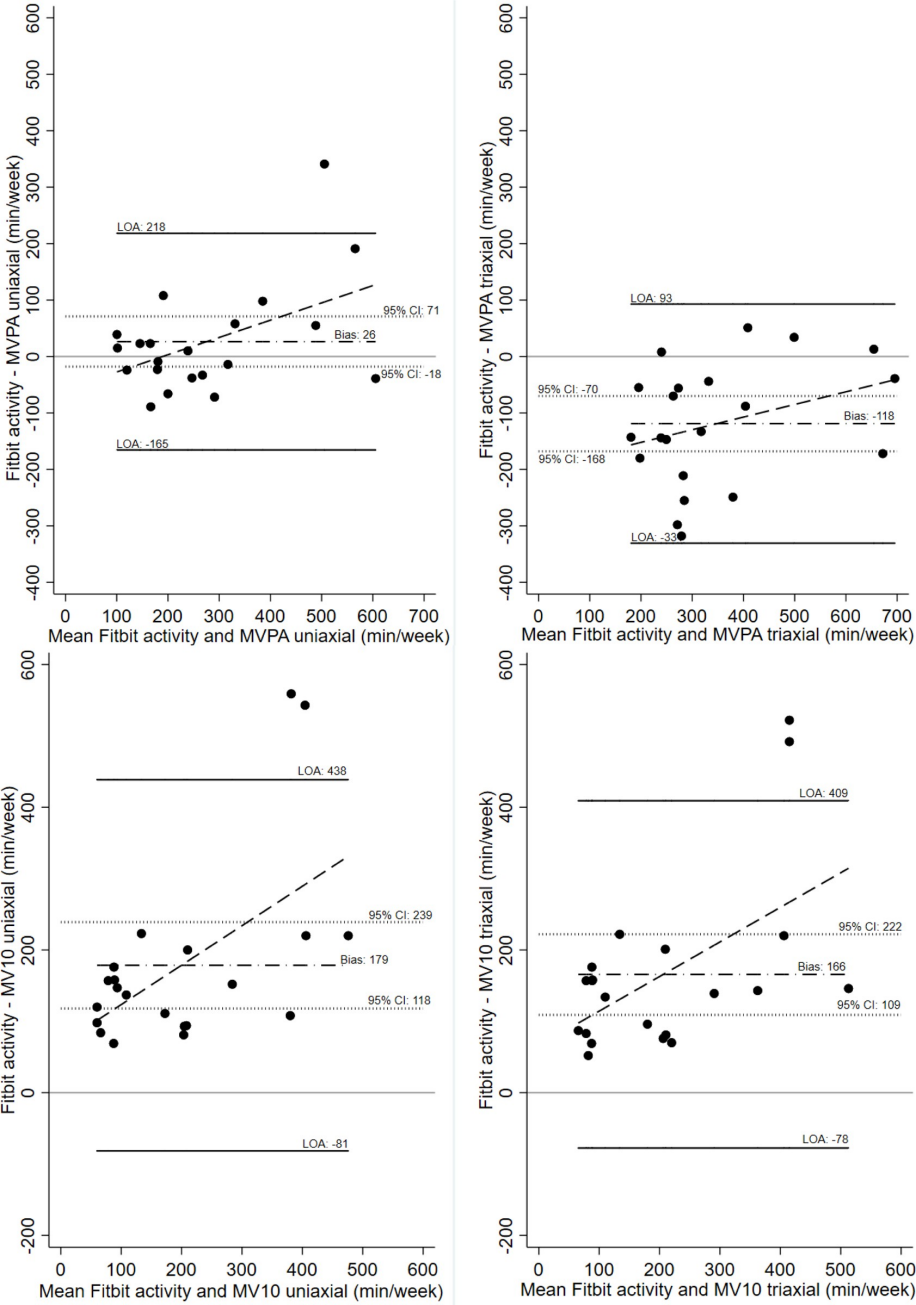
Bland Altman plots to indicate agreement between Fitbit and Actigraph derived moderate-to-vigorous physical activity. Bland Altman plots for Fitbit and Actigraph Moderate-to-Vigorous Physical Activity (MVPA) (top) and Fitbit and MVPA in bouts of ≥10-minutes (MV10) (bottom) agreement for uniaxial (left) and triaxial (right) defined activity. LOA: limits of agreement; 95% CI: 95% confidence interval for the mean difference; bias: mean difference.

The relationship between Fitbit measured active minutes and Actigraph-measured MVPA (top) and MV10 (bottom) for both uniaxial and triaxial cut points at week-12 are shown in Fig 4. For this analysis only data from participants who engaged with their Fitbit during the time they were also wearing the Actigraph at the end of the intervention phase were included (n = 21). Fitbit and Actigraph activity measures were significantly correlated in all cases (with Spearman r correlations between 0.60 and 0.91 for MVPA using triaxial and uniaxial cut-points). The correlations are also displayed in Fig 4. The agreement between these measures, however, was poor (see Bland Altman plots in Fig 5). Fitbit consistently measured more activity than Actigraph MV10 (uniaxial and triaxial) but recorded less activity than Actigraph MVPA using triaxial cut points. Fitbit measured activity was closest to MVPA measured using the uniaxial definition as no bias was observed in this case (i.e. the 95% CI included zero). Although most differences fell within the 95% limit of agreement (LOA; Fig 5), the width of all LOAs was very wide, and increasing differences in activity measured by the two devices was associated with increasing means.

## Discussion

Given the concerns regarding sustained engagement with wearable activity trackers [22], it is important to explore sustained engagement with trackers and patterns of PA over time, and especially once intervention supports have been removed. To our knowledge, this is the first study to explore wearable tracker wear-time throughout the intervention and maintenance period of a RCT.

Consistent with previous research [20, 21], Fitbit adherence was high and steady during the intervention. Our study is one of the first to demonstrate that Fitbit adherence was also high during the 12-week maintenance phase. Participant daily step count was maintained in the follow-up phase. However, Fitbit derived active minutes decreased during the maintenance period. Cadmus-Bertram and colleagues [20], in their study of overweight post-menopausal women also reported a larger decline in active minutes by the end of intervention (16-weeks) compared to steps (14% vs 8% reduction) respectively. The maintenance of steps and simultaneous non-significant decline in active minutes over the course of our study is interesting. A likely explanation is that participants are more likely to focus on steps rather than active minutes and are much more aware of the 10,000 steps per day message than they are the recommendations concerning MVPA and the importance of exercise intensity [32]. Overall, the high adherence to Fitbit wear over the 24-weeks displays promise as a behaviour change tool. This contrasts with two recent observational studies reporting declines in Fitbit Zip and Garmin Vivosmart use over the course of a year and 7-months respectively [33, 34]. In the Study by Grym et al. [34] wear-time decreased as pregnancy progressed and particularly following giving birth. One possible explanation for the differences concerning Fitbit use in our study compared to the first study [33] is the device used. The observational study used the Fitbit Zip, a device that is clipped on to clothing. Therefore, it likely requires further effort to use it because it must be repeatedly taken off and on. In contrast, the Fitbit Alta is a wrist-worn device that does not need to be removed (except for showering or swimming) and the data is easier to view in real-time. The second study [34] used a similar tracker in specification and the mean daily step count was only 5576 compared to over 8000 in the present study. It is unlikely that a smart wearable device alone is sufficient to produce sustained increases in physical activity in the long-term unless part of a more comprehensive intervention, which may explain the higher adherence and sustained step count in the present study. We did not find evidence to support an influence of staff contact on activity levels. However, as this is a secondary analysis and comprises a small sample of participants, further work is needed to determine the role of staff contact in activity level change.

There was no association between Fitbit wear-time and Actigraph-derived MVPA across the intervention period. This contrasts with previous findings that greater adherence to wearing the Fitbit was associated with greater increases in ActiGraph-measured MVPA [21]. However, it should be noted that the adherence rates in this part of the study were relatively extreme if not bimodal, with clusters of scores close to 0 or 100%, with few examples of more ‘moderate’ rates. To what extent this reflects population behaviour (i.e. all people tend to use a Fitbit very regularly or not at all) or a consequence of the small sample size is not known, and targeted studies with larger samples will be needed in order to elucidate the relationship between adherence and behaviour change.

Fitbit-measured active minutes and Actigraph-derived MVPA and MV10 were significantly positively correlated at both 12-weeks and 24-weeks. However, the agreement between these measures was poor with the Fitbit consistently reporting more activity than Actigraph derived MV10 (uniaxial and triaxial), but less activity than Actigraph derived MVPA using triaxial cut points. Despite being highly correlated, the Bland Altman plots indicate that the level of agreement between the Fitbit Alta and the Actigraph is insufficient to be interchangeable. However, Fitbit measured active minutes was closest to Actigraph- derived MVPA using uniaxial cut points as no bias was observed in this case. The substantial bias in recording higher active minutes for the Fitbit Alta is likely due to the fact that active minutes is unable to account for exercise intensity and therefore any light activity performed for at least 10-minutes at a time may have been counted as ‘active minutes’. Therefore, there is a likelihood of the Fitbit Alta overestimating true MVPA.

### Study limitations

The study is limited by a relatively small sample size. The analysis concerning the agreement between the Fitbit and Actigraph is exploratory and was only based on a subset of the intervention group (*n* = 22) because missing data from either of the devices precluded inclusion of more participants. A further limitation is the use of a Fitbit device without heart rate, limiting applicability to other smart wearables that mostly include a heart rate monitor.

### Clinical implications

Given the resource implications of using accelerometers in clinical trials, and the capacity to easily monitor wearable tracker derived PA data in the long-term, future work should continue to assess the level of agreement between devices in order to find convenient and accessible ways to monitor PA. Further work would do well to explore the use of wearable activity trackers that measure activity or intensity minutes using heart rate and age-related intensity zones and compare these measures to that of accelerometer derived MVPA.

## Conclusion

This is one of the first studies to explore wearable tracker wear-time and activity patterns throughout a trial including the maintenance phase. The study found wearable-tracker wear time to be high throughout the 24-week trial. Step count was maintained throughout the trial displaying promise for the effectiveness of smart-wearable interventions to reduce sedentary behaviour beyond the intervention period. Further worthwhile work could compare more advanced smart-wearable technology with accelerometers to improve agreement between the measures and explore less resource-intensive methods to assess PA that could be scalable. Further work exploring interaction with wearable technology (App and watch) and activity monitoring could also be worthwhile to better understand the facilitators and barriers to sustained engagement with wearable technology, and in turn, long-term PA participation.

## Supporting information

S1 Table Median difference (IQR) in steps per day per week, and active minutes per week, compared with study staff contact week 4, 8 and 12. (DOCX)

S1 Fig (DOCX)
