# Supplementary material for: Fitbit wear-time and patterns of activity in cancer survivors throughout a physical activity intervention and follow-up: Exploratory analysis from a randomised controlled trial
Source: PLoS One. 2020 Oct 19;15(10):e0240967. doi: 10.1371/journal.pone.0240967 (PMC7571692; doi:10.1371/journal.pone.0240967)
Supplement: S1 Table — (DOCX) [file pone.0240967.s001.docx]

Supplement Table S1. Median difference (IQR) in steps per day per week, and active minutes per week, compared with study staff contact week 4, 8 and 12.

| Weeks | Steps per day per week | | | Active minutes per week | | |
| --- | --- | --- | --- | --- | --- | --- |
|  | Median difference (IQR) | z^1^ | p | Median difference (IQR) | z^1^ | p |
| 3 v 4 | -204  (-1580 to 731) | 0.70 | 0.486 | -23.4 (-78 to 29) | 1.44 | 0.149 |
| 4 v 5 | -604 (-1256 to 573) | 1.36 | 0.174 | -10 (-76 to 19) | 0.88 | 0.382 |
| 7 v 8 | -345 (=1465 to 540) | 1.14 | 0.253 | -50.1 (-107 to 27) | 1.91 | 0.056 |
| 8 v 9 | -111 (-599 to 1019) | -0.83 | 0.407 | 19 (-36 to 74) | -1.13 | 0.259 |
| 11 v 12 | 138 (-833 to 1867) | -0.57 | 0.473 | -1 (-82 to 112) | 0.04 | 0.972 |
| 12 v 13 | 99 (-525 to 968) | -0.75 | 0.455 | 20 (-61 to 86) | -0.61 | 0.543 |

^1^ Z is the normal approximation of the Wilcoxon signed rank test statistic. IQR is the interquartile range.
