# Supplementary figures and images for: Fitbit wear-time and patterns of activity in cancer survivors throughout a physical activity intervention and follow-up: Exploratory analysis from a randomised controlled trial
Source: PLoS One. 2020 Oct 19;15(10):e0240967. doi: 10.1371/journal.pone.0240967 (PMC7571692; doi:10.1371/journal.pone.0240967)

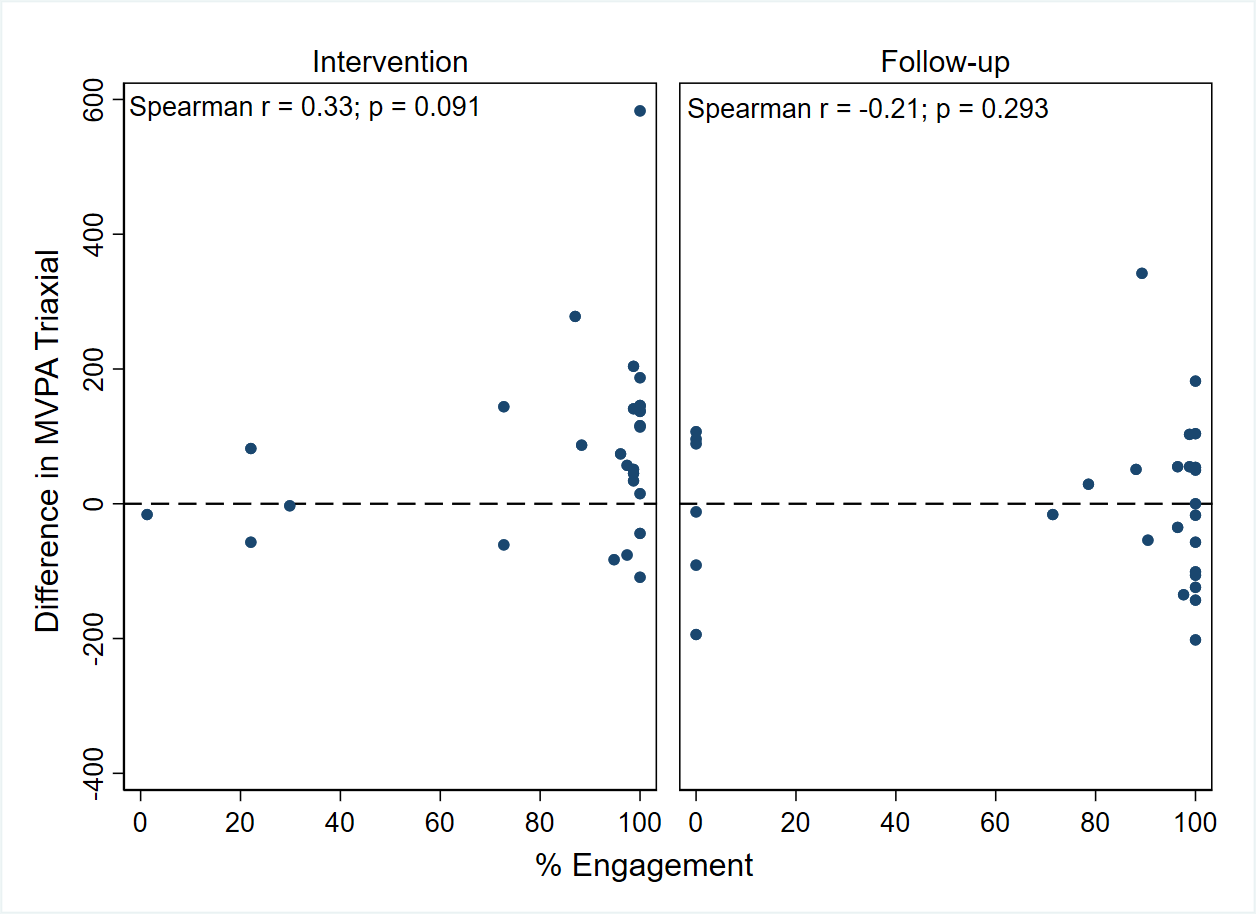


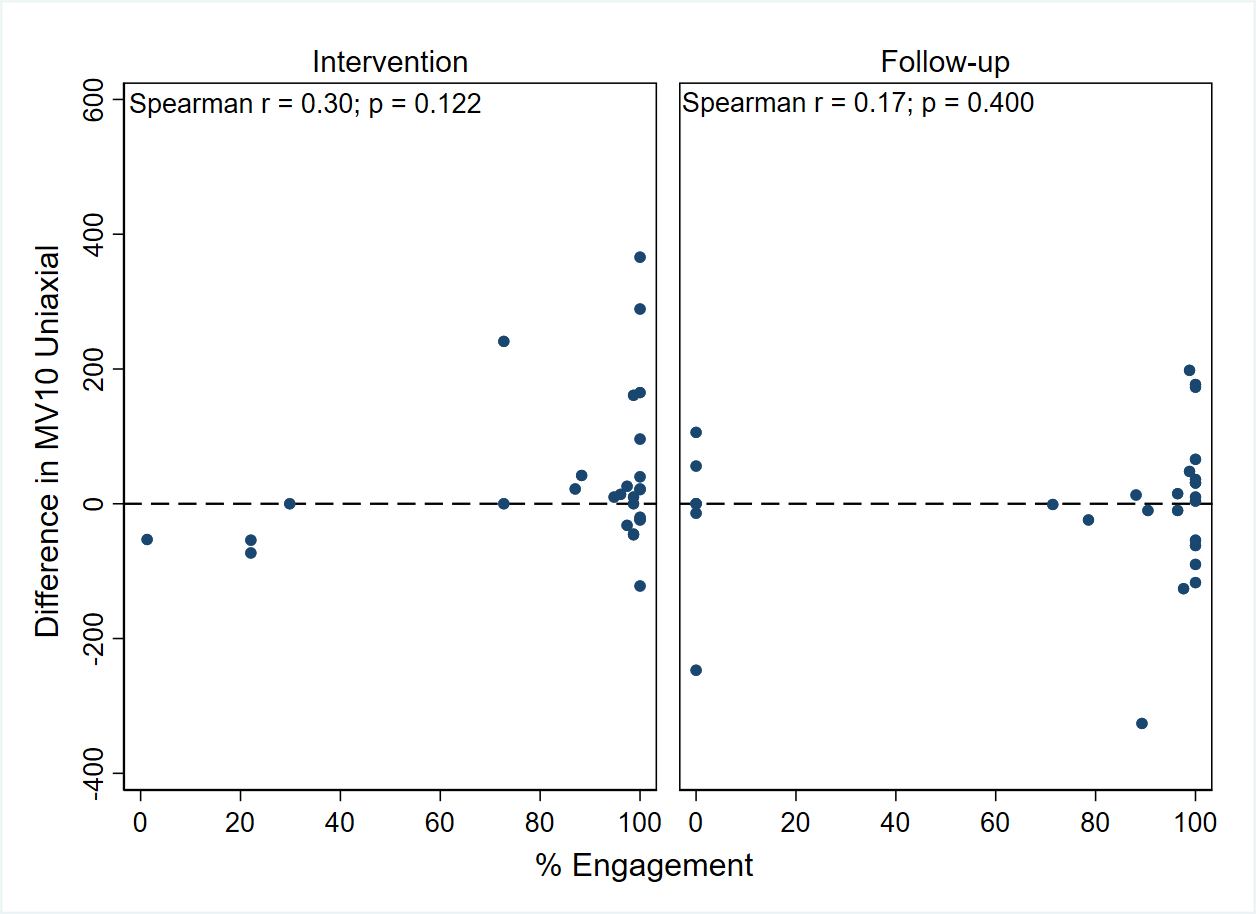


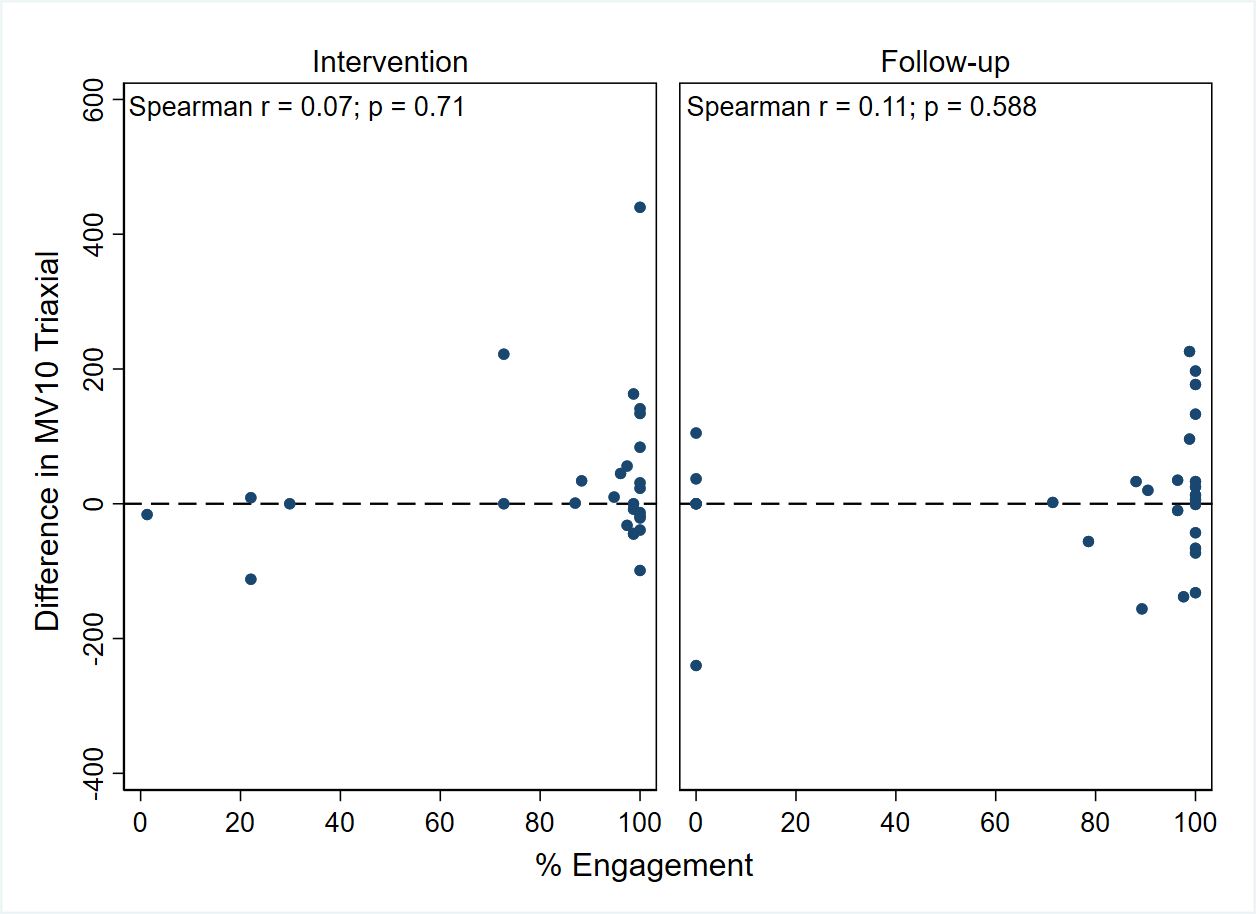

Supplement: S1 Fig — (DOCX) [file pone.0240967.s002.docx]
